# Distribution and impact on quality of life of the pain modalities assessed by the King’s Parkinson’s disease pain scale

**DOI:** 10.1038/s41531-017-0009-1

**Published:** 2017-03-15

**Authors:** Pablo Martinez-Martin, Jose Manuel Rojo-Abuin, Alexandra Rizos, Carmen Rodriguez-Blazquez, Claudia Trenkwalder, Lauren Perkins, Anna Sauerbier, Per Odin, Angelo Antonini, Kallol Ray Chaudhuri, on behalf of KPPS, EUROPAR and the IPMDS Non Motor PD Study Group

**Affiliations:** 1 National Center of Epidemiology and CIBERNED, Carlos III Institute of Health, Madrid, Spain; 2 Department of Statistics, Center of Human and Social Sciences, Spanish Council for Scientific Research, Madrid, Spain; 3 King’s College Hospital, London, UK; 4 Paracelsus-Elena Hospital, Kassel, Germany; 5 King’s College London, London, UK; 6 University of Lund, Lund, Sweden; 7 Klinikum Bremerhaven Reinkenheide, Bremerhaven, Germany; 8 Parkinson and Movement Disorders Unit IRCCS Hospital San Camillo, Venice, Italy; 9 University Hospital Lewisham, London, UK

## Abstract

In Parkinson’s disease, pain is a prevalent and complex symptom of diverse origin. King’s Parkinson’s disease pain scale, assesses different pain syndromes, thus allowing exploration of its differential prevalence and influence on the health-related quality of life of patients. Post hoc study 178 patients and 83 matched controls participating in the King’s Parkinson’s disease pain scale validation study were used. For determining the respective distribution, King’s Parkinson’s disease pain scale items and domains scores = 0 meant absence and ≥1 presence of the symptom. The regular scores were used for the other analyses. Health-related quality of lifewas evaluated with EQ-5D-3L and PDQ-8 questionnaires. Parkinson’s disease patients experienced more pain modalities than controls. In patients, Pain around joints (King’s Parkinson’s disease pain scale item 1) and Pain while turning in bed (item 8) were the most prevalent types of pain, whereas Burning mouth syndrome (item 11) and Pain due to grinding teeth (item 10) showed the lowest frequency. The total number of experienced pain modalities closely correlated with the PDQ-8 index, but not with other variables. For all pain types except Pain around joints (item 1) and pain related to Periodic leg movements/RLS (item 7), patients with pain had significantly worse health-related quality of life. The influence of pain, as a whole, on the health-related quality of life was not remarkable after adjustment by other variables. When the particular types of pain were considered, adjusted by sex, age, and Parkinson’s disease duration, pain determinants were different for EQ-5D-3L and PDQ-8. King’s Parkinson’s disease pain scale allows exploring the distribution of the diverse syndromic pain occurring in Parkinson’s disease and its association with health-related quality of life.

## Pain: Under-appreciated impact on quality of life

Researchers confirm that the pain experienced by patients with Parkinson disease (PD) is a key determinant of health-related quality of life. Pain is a common non-motor symptom that is often underacknowledged and undertreated. Using data from the recently validated pain scale for PD, Pablo Martinez-Martin (Carlos III Institute of Health in Madrid, Spain) and colleagues explored the impact of specific pain modalities on patients’ quality of life. They found that patients with PD reported experiencing twice as many types of pain than controls, with pain around the joints and pain while moving in bed being the most prevalent. Furthermore, there was a close correlation between the number of experienced pain modalities and quality of life as evaluated with the PD questionnaire PDQ-8. Understanding the different types of pain in PD will aid the provision of effective pain relief and greatly improve patients’wellbeing.

## Introduction

Pain is a common non-motor symptom of Parkinson’s disease (PD), frequently undeclared and, consequently, undertreated.^1, 2^ Nowadays, it is well known that different types of pain can be recognized in PD patients,^3, 4^ a fact that makes appropriate assessment and management of this symptom difficult. Although several studies showed a deleterious effect of pain on the health-related quality of life (HRQoL), the specific effect of pain on quality of life in people with PD has not been tested with specific pain measures and is partially unclear.^5, 6^

HRQoL refers to those aspects of the individuals’ quality of life (QoL) related with health status and care and, therefore, is a more restricted concept than ‘global QoL’. As there is no a universally accepted definition of HRQoL, we define here this construct as: “the perception and evaluation, by patients themselves, of the impact caused on their life by the disease and its consequences”.^7^

Determinant factors (e.g., depression, disability, and insomnia) influence the HRQoL and, therefore, the association between these factors and the HRQoL is close and consistent. On the other hand, modification of the determinant factors will result in changes of the HRQoL. Thus, their identification and appropriate management may be crucial for improving the patients’ QoL. Pain is a widely recognized determinant of QoL in any setting and also in PD,^6, 8^ a condition in which pain is a complex and highly prevalent symptom.^9^

Recently, the King’s Parkinson’s Disease PainScale (KPPS) has been validated as the first specific rating scale to evaluate the burden of pain in the context of PD. The KPPS assesses seven different domains corresponding to the diverse modalities of pain identified in PD. In the first validation study, a high correlation was found between the KPPS total score and the summary indexes of a generic (EQ-5D-3L) and a PD-specific (PDQ-8) HRQoL instrument.^10^

Taking advantage of the existing data from the validation study and the close relationships between pain burden and QoL, we explored in the present study the distribution in the sample of the different types of pain assessed by the KPPS and how they impact on the HRQoL of PD patients.

## Results

The characteristics of the sample are shown in Table 1S (Supplementary material). Most of patients (71.35%) were in intermediate motor stages of disease (HY 2 and 3). Table 1 shows the results obtained with the measures applied in the study.

**Table 1:** Descriptive statistics of the assessments in the study

	Patients				Controls			
	Mean	SD	Med.	Range	Mean	SD	Med.	Range
SCOPA-motor scale	17.38	10.28	15	1–65	–	–	–	–
NMSS	60.71	44.31	48	0–235	–	–	–	–
CISI-PD	6.54	3.93	6	0–19	–	–	–	–
HADS-anxiety	6.17	4.56	5	0–20	5.92	4.32	5	0–20
HADS-depression	5.44	3.96	5	0–18	4.69	3.81	4	0–18
EQ-5D-3L	0.52	0.28	0.62	−0.43–1	0.60	0.29	0.66	−0.43–1
PDQ-8	27.84	20.28	25	0–93.75	–	–	–	–
PDSS-2	18.25	11.20	16.50	0–51	–	–	–	–
VAS pain	32.92	23.84	27.67	0–100	–	–	–	–
King’s PD pain scale								
1. Musculoskeletal pain	6.02	4.07	6	0–12	3.66	3.69	3	0–12
2. Chronic pain	3.37	5.53	0	0–24	1.37	3.23	0	0–18
3. Fluctuation-related pain	5.27	8.26	0	0–36	0.63	2.30	0	0–14
4. Nocturnal pain	4.91	5.87	3	0–24	1.49	3.36	0	0–15
5. Oro-facial pain	0.97	3.00	0	0–22	0.24	1.42	0	0–12
6. Discolouration, OS	2.29	4.49	0	0–24	0.87	2.66	0	0–15
7. Radicular pain	2.36	3.53	0	0–12	1.07	2.49	0	0–12
KPPS Total score	25.19	22.14	17	0–102	9.34	12.58	4	0–63

*SD* standard deviation, *Med* median, *SCOPA* scales for outcomes in Parkinson’s disease, *NMSS* non-motor symptoms scale, *CISI-PD* clinical impression of severity index-Parkinson’s disease, *HADS* hospital anxiety and depression scale, *EQ-5D-3L* EuroQoL-5 dimensions-3 levels, *PDQ-8* Parkinson’s disease questionnaire-8 items, *PDSS-2* Parkinson’s disease sleep scale-version 2, *VAS* visual analog scale. Discolouration, *OS* discolouration; edema/Swelling, *KPPS* King’s Parkinson’s disease pain scale

In Table 2, the frequency in the sample of the diverse modalities of pain is shown. PD patients had 4.0 (SD = 2.9) types of pain in average, whereas controls had 2.0 (SD = 2.0) (*p* < 0.0001). Four or more different types of pain were declared by 48.2% of patients and by 19.3% of controls (*p* < 0.0001). The most frequently declared type of pain was pain around joints (KPPS item 1), followed by pain while turning in bed (item 8). On the contrary, the lowest prevalence in the series was for burning mouth syndrome (item 11) and pain due to grinding teeth, bruxism (item 10). Only one patient (0.56%) scored “no pain” on the KPPS and no patient scored on all the fourteen items, with the maximum accumulation of pain modalities (13 items scored positively) observed in only one patient (0.56%). All types of pain were more prevalent in PD patients than in controls and the most significant differences were observed for pain related with dyskinesias, fluctuations, and turning in bed (all three, *p* ≤ 0.003). In controls, 19.3% reported no pain and one subject (1.2%) scored in up to nine different types of pain. Ninety eight patients (55.1%) and 25 controls (30.1%) (*p* = 0.0002) were receiving analgesic medications.

**Table 2:** Types of pain prevalence in the sample

	Prevalence (%)		
King’s Parkinsons’ disease pain scale items	PD patients	Controls	*p*
Domain 1: musculoskeletal pain			
1. Pain around joints	84.8	69.9	0.005
Domain 2: chronic pain			
2. Pain deep within the body	31.5	18.1	0.023
3. Pain related to internal organ	23.0	14.5	0.11
Domain 3: fluctuation-related pain			
4. Dyskinetic pain	18.5	4.8	0.003
5. “Off” dystonia in a region	33.2	6.0	<0.0001
6. Generalized “off” period pain	25.8	4.8	0.0001
Domain 4: nocturnal pain			
7. PLM or RLS-associated pain	29.2	13.3	0.005
8. Pain while turning in bed	48.3	16.9	<0.0001
Domain 5: Oro-facial pain			
9. Pain when chewing	8.4	1.2	0.023
10. Pain due to grinding teeth	7.3	2.4	0.11
11. Burning mouth syndrome	5.1	1.2	0.13
Domain 6: discolouration; edema/swelling			
12. Burning pain in the limbs	21.9	9.6	0.016
13. Lower abdominal pain	19.1	8.43	0.027
Domain 7: radicular pain			
14. Shooting pain/pins and needles	46.1	28.9	0.008

Benjamini–Hochberg correction for multiple comparisons: for prevalence (*m *= 14): *p *< 0.026.

*PD* Parkinson’s disease, *PLM* periodic leg movements, *RLS* restless legs syndrome

In PD patients, only musculoskeletal pain was significantly more frequent in women (97% vs. 77.7%; *p* < 0.001). Modalities of nocturnal pain (dyskinetic and pain related with “off dystonia”) tended to decrease with age, but did not reach statistical significance after correction for multiple testing (*p* < 0.025 needed). Items 5 and 6 (fluctuation-related pains) and item 14 (radicular pain), however, were significantly less frequent in patients with higher age at onset of PD (*p* = 0.002–0.015). The three components of the nocturnal pain domain were significantly more frequent in patients with longer disease duration (*p* = 0.014 to <0.001). No other significant differences were observed regarding these variables.

The total number of experienced syndromic pain was not statistically different by gender (3.8, men; 4.4, women; *p* = 0.10). This total number correlated moderately with SCOPA-Motor (*r*_S_ = 0.38), clinical impression of severity index for PD (CISI-PD) (*r*_S_ = 0.37) and EQ-5D-3L Index (*r*_S_ = −0.41), and highly with the PDQ-8 Index (*r*_S_ = 0.55) (all coefficients, *p* < 0.001). Correlation values with age, years of education, age at onset, PD duration, HY, and levodopa-equivalent daily dose were negligible or weak (*r*_S_ < 0.30).

The difference in HRQoL indexes between patients with and without the diverse pain modalities is displayed in Table 3. Differences were significant, even after correction for multiple comparisons, for all items except items 1 (Pain around joints) and 7 (Periodic leg movements or Restless legs syndrome-associated pain). To be highlighted, both HRQoL indexes showed similar trends in this analysis, although differences between them were observed. The correlation of the HRQoL indexes with the KPPS dimensions and total score are shown in Table 2S (Supplementary material). As a whole, the strength of the association with the KPPS items was weak, with some items showing moderate values with the EQ-5D-3L (KKPS item 1; *r*_S_ = 0.36; *p* < 0.001) and PDQ-8 indexes (KPPS items 3, 12, 13, and 14; *r*_S_ = 0.35–0.41; *p* < 0.001). Coefficient values indicated a close and similar association between the KPPS total score and both HRQoL indexes.

**Table 3:** Types of pain and quality of life indexes in Parkinson’s disease patients

King’s Parkinsons’ disease pain scale items	EQ-5D-3L			PDQ-8		
	No	Yes	*p*	No	Yes	*p*
1. Pain around joints	0.59 ± 0.25	0.51 ± 0.29	0.1	25.11 ± 19.62	28.33 ± 20.42	0.43
2. Pain deep within the body	0.57 ± 0.23	0.41 ± 0.35	0.006	24.18 ± 18.72	35.83 ± 21.40	0.0004
3. Pain related to internal organ	0.55 ± 0.26	0.41 ± 0.33	0.005	24.06 ± 18.85	40.47 ± 20.00	<0.0001
4. Dyskinetic pain	0.57 ± 0.25	0.31 ± 0.32	<0.0001	24.91 ± 19.53	40.72 ± 18.68	<0.0001
5. “Off” dystonia in a region	0.55 ± 0.29	0.46 ± 0.27	0.007	25.03 ± 20.76	33.53 ± 18.15	0.001
6. Generalized “off” period pain	0.57 ± 0.25	0.39 ± 0.33	0.0005	24.53 ± 18.30	37.36 ± 22.76	0.0008
7. PLM or RLS-associated pain	0.54 ± 0.28	0.48 ± 0.30	0.13	25.79 ± 19.48	32.81 ± 21.49	0.038
8. Pain while turning in bed	0.58 ± 0.26	0.45 ± 0.29	0.0001	20.18 ± 17.56	36.05 ± 19.87	<0.0001
9. Pain when chewing	0.54 ± 0.28	0.35 ± 0.31	0.017	26.23 ± 19.50	45.42 ± 20.99	0.001
10. Pain due to grinding teeth	0.53 ± 0.28	0.37 ± 0.32	0.011	26.61 ± 19.48	43.51 ± 24.39	0.011
11. Burning mouth syndrome	0.53 ± 0.28	0.34 ± 0.32	0.014	26.26 ± 19.35	57.64 ± 13.63	0.0001
12. Burning pain in the limbs	0.55 ± 0.26	0.40 ± 0.33	0.001	23.99 ± 18.44	41.59 ± 20.81	<0.0001
13. Lower abdominal pain	0.55 ± 0.27	0.39 ± 0.32	0.0004	24.76 ± ± 19.73	40.90 ± 17.38	<0.0001
14. Shooting pain/pins and needles	0.56 ± 0.27	0.47 ± 0.30	0.01	21.88 ± 18.08	34.83 ± 20.58	<0.0001

Benjamini–Hochberg correction for multiple comparisons: (*m* = 28): *p* < 0.025, where ‘*m*’ is the number of tests considered

*PDQ-8* Parkinson’s disease questionnaire-8 items, *PLM* periodic leg movements, *RLS* restless legs syndrome

The results of the multiple regression models are shown in Table 4. In the phase 1, the influence of pain (KPPS) on the generic HRQoL (EQ-5D-3L Index) was just at the limit of the statistical significance after controlling for the other factors in the model, with functional state (SCOPA-Motor ADL) and depression (HADS-Depression) as the only significant variables influencing the generic HRQoL. For the model with the specific PDQ-8, only depression, ADL, and sleep (PDSS-2) were significant determinants. For the phase 2 models, musculoskeletal pain, fluctuation-related pain, and PD duration were the factors significantly and independently influencing the EQ-5D-3L Index, whereas nocturnal pain, peripheral discolouration/oedema related pain, and (again) PD duration were the key factors for the PDQ-8 index.

**Table 4:** Results of the multiple linear regression models

	Adjusted *R* ^2^	Coeff.	SE	Significance	Beta
Model 1					
EQ-5D-3L index	0.43				
SCOPA-ADL		−0.022	0.007	0.001	−0.28
HADS-Depression		−0.013	0.005	0.019	−0.18
KPPS		−0.002	0.001	0.047	−0.17
Model 2					
PDQ-8 index	0.67				
HADS-Depression		2.51	0.29	<0.001	0.49
SCOPA-ADL		1.43	0.36	<0.001	0.26
PDSS-2		0.40	0.11	<0.001	0.22
Model 3					
EQ-5D-3L index	0.35				
Musculoeskeletal		−0.018	0.005	<0.001	−0.26
Fluctuation-related		−0.008	0.003	0.002	−0.24
PD duration		−0.012	0.004	0.003	−0.21
Model 4					
PDQ-8 index	0.31				
Nocturnal		0.68	0.26	0.01	0.20
Discolouration, edema		0.81	0.38	0.03	0.18
PD duration		0.70	0.29	0.02	0.17

*Coeff*. coefficient, *SESCOPA-ADL* scales for outcomes in Parkinson’s disease-activities of daily living, *HADS* hospital anxiety and depression scales, *KPPS* King’s Parkinsons’ disease pain scale, *PDSS-2* Parkinson’s Disease Sleep Scale-Version 2, *PD* Parkinson’s disease, *PDQ-8* Parkinson’s disease questionnaire-8 items

## Discussion

Pain has been recognized in 40–85% of PD patients,^3, 9^ and is a complex manifestation in this condition that expresses a variety of syndromic pain, including musculoskeletal and visceral nociceptive pain, central and peripheral neuropathic pain, and other modalities. Apart from this, 25–64% of PD patients are thought to experience pains unrelated to this disorder.^3^

This study was carried out on a sample of PD patients systematically characterized by declaring otherwise unexplained pain, without dementia or recognized disorders causing pain, and matched controls. Using the KPPS, it was possible to explore the distribution of the different modalities of pain assessed by this scale in both groups and the main findings were:

1. The average number of pain types present in PD patients (*n* = 4) was double than in controls (*n* = 2). The maximum number of pain modalities experienced was significantly higher in the patients group (13/14 vs. 9/14).
2. In both groups, the most prevalent modality of pain was musculoskeletal pain, whereas the lowest were the oro-facial pains.
3. Nocturnal pain (pain while turning in bed and pain related to periodic leg movements/restless legs syndrome) was significantly more prevalent in patients.

Musculoskeletal pain may be a dominant symptom in early PD stages and has been attributed the cause of 40–90% of the reported pain, as well as the most prevalent type (41–70%), followed by dystonic pain (40–48%), radicular-neuropathic pain (14–35%), central neuropathic pain (22–36%), and other modalities pains (5.7%).^4, 8, 9, 11^ The corresponding figures for the present study were: musculoskeletal, 84.8%; dystonic, 33.2%; radicular, 46.1%; central neuropathic, 31.5%; and oro-facial pain, 20.8%. The origin of musculoskeletal pain in PD is complex and is a mixture of nociceptive and neuropathic elements complicated by local joint related pain as well as parkinsonian rigidity and postures such as dystonia. The recently reported PANDA study for instance, reported efficacy of the active drug (oxycodone/naloxone combination) on musculoskeletal pain when applying the KPPS.^12^ Thus, reports of pain symptoms being dominated by musculoskeletal element is not surprising and is consistent with published data.

In a study, chronic pain was found in 61.8% of PD patients declaring pain,^13^ whereas in the present study (KPPS items 2 and 3) was present in 54.5%. In the same vein, it has been found that 74–82% of patients had three or less types of pain,^3, 14^ whereas this proportion was 51.8% in the present study. Differences can be explained by the selection of patients for the present study and by the content of the KPPS, which explores modalities of pain not included in other rating scales, further confirming the need for the use of validated specific instruments such as the KPPS. The proportion of PD patients receiving analgesics was 55.1% in our study, a figure quite close to the 52.4% found by Broen et al.^9^

Although the prevalence and severity of pain in PD has been found to be higher in women,^3, 15–17^ this finding is not universal.^18, 19^ In the present study, the sum of different types of pain was mildly higher in women, but the difference was not statistically significant. The correlations between number of experienced types of pain and other variables in the study were low or moderate as a whole and lower than the correlations between severity of pains and those variables.^10^ The only close association between number of different types of pain and other constructs in the study was observed with the PDQ-8 Index (*r*_S_ = 0.55). Other studies have found relationships between pain and motor complications^13, 20^ or disease progression,^21^ but no with factors like age at diagnosis, disease duration, motor examination, or PD stage.^3, 19–21^

At any setting, there is a wide range of factors influencing HRQoL as, for example, depression, disability, sleep disorders, and pain. In PD many of these determinant factors are present, frequently in a combined and variable manner, so that personalized analysis is needed to identify the most important ones and establish the priority order for intervention. Pain has been identified as a major correlate and a determinant factor of HRQoL in PD.^6, 15, 22–25^ Nonetheless, this finding is not uniform and several studies did not report an effect of pain on the HRQoL in PD patients. For instance, Schrag et al. found no difference between the U.K. general population and patients with PD on the relationship between pain and HRQoL^5^ and pain did not appear among the HRQoL determinants in other studies.^26^ However, characteristics of the samples (age, disease duration, and education level) and instruments applied for measuring both pain and HRQoL in these studies differ from the present one. Furthermore, a specific validated instrument for assessment of pain in PD was not used in these studies.

In the present study, we have found that most of the pain modalities included in the KPPS have an effect on HRQoL, with exception to musculoskeletal pain and pain related to periodic leg movements/restless legs syndrome (Table 3). Pain, represented by the total KPPS score, however, did not appear as a determinant of HRQoL or showed only a modest effect after adjustment by other variables (Table 4). In the study by Rahman et al.^22^ the presence or absence of pain, globally considered, did not condition a significant difference on HRQoL estimated with the PDQ-39, although showed an effect in the regression analysis. Gallagher et al.^23^ using a visual analog scale for measuring pain, observed a high correlation with the PDQ-39 index, although this relationship disappeared after adjustment with motor and non-motor variables. A similar fact, although in contrary direction (appearing as determinant only after adjustment by other variables) was observed with musculoskeletal pain for the EQ-5D-3L in the present study. Obviously, the severity of other symptoms and the importance of other concurrent factors can moderate the influence of pain on the QoL.

Finally, concerning the influence of the different types of pain, musculoskeletal and fluctuation-related domains showed a significant independent effect on the EQ-5D-3L model, whereas nocturnal pain and discolouration/edema were significant for the PDQ-8 model. In addition, PD duration appeared as an independent influencing factor in both models highlighting the prominence of the disease progression over time on the patients’ HRQoL. Interestingly, two studies have now reported the efficacy of dopaminergic (rotigotine patch)^27^and non-dopaminergic (oxycodone with naloxone)^12^ agents on pain in randomised placebo-controlled studies. Using the KPPS, the dominant pains responding to the active agents were fluctuation related pain (rotigotine) and musculoskeletal pain (oxycodone with naloxone) and thus a tangible effect of these therapies on HRQoL could be envisaged.^12, 27^

Limitations of this study are: (1) the study was not specifically designed for investigating the prevalence of pain types in PD population; (2) patients included in the study were selected on the basis of declaring pain of undetermined origin; therefore, the sample is biased (selection bias) and the distribution of pain types cannot be extrapolated to the PD population; (3) few data on pain severity are included in the present study, as most of them were explored in the pivotal KPPS validation study.^10^

This study is the first application of the KPPS to a sample of patients with PD and pain, and provided the opportunity of starting epidemiological analyses offering new data on the distribution and relationships of the diverse pain types occurring in PD and its links with HRQoL.

## Methods

### Design

Post hoc study of the first KPPS validation study.^10^

### Patients

One hundred seventy eight patients with a diagnosis of idiopathic PD according to the UK PD Brain Bank criteria^28^ experiencing otherwise unexplained pain as declared in item 10 of the NMS Questionnaire^29^ were included. Exclusion criteria were (1) diagnosis of parkinsonism different to idiopathic PD; (2) dementia (as per internationally accepted criteria); (3) disorders causing pain unrelated to PD (e.g., severe osteoarthritis, malignancy); and (4) inability to provide consent to participate in the study.

Controls data were obtained from the KPPS validation study cohort. A ratio of patients to controls of 2:1 was estimated and 83 non-spousal, non-PD, age- and sex-matched controls were included.^10^

Recruitment of patients and controls was carried out from December 2012 to April 2014.

### Assessments

In addition to recording sociodemographic data and medical history, the following assessments were applied:

1. The KPPS,^10^ a rater-interview-based scale with the patient (helped by the caregiver if needed) addressed to determine localization, intensity, and frequency of pain and its relationships with motor fluctuations or musculoskeletal pain. The KPPS is composed of 14 items divided into seven domains: 1. Musculoskeletal pain (item 1); 2. Chronic pain (items 2 and 3); 3. Fluctuation-related pain (items 4–6); 4. Nocturnal pain (items 7 and 8); 5. Orofacial pain (items 9–11); 6. Discoloration, Edema/Swelling (items 12 and 13); and 7. Radicular pain (item 14). Each item is scored by severity (0, none to 3, very severe) multiplied by frequency (0, never to 4, all the time), with a range from 0 to 12. A total KPPS score is obtained from the sum of the items’ scores (theoretical range: 0–168) and represents the symptomatic burden by pain.
2. Clinician-based evaluations: Hoehn and Yahr (HY) staging;^30^ scales for outcomes in PD-Motor (SCOPA-Motor);^31^ non-motor symptoms scale;^32^ and CISI-PD.^33^
3. Patient-reported outcomes: hospital anxiety and depression scale (HADS);^34^ EQ-5D-3L, a generic, preference-based HRQoL measure;^35^ PDQ-8, a specific instrument for assessment of HRQoL in PD;^36^ PD sleep scale-version 2 (PDSS-2);^37^ QUICK wearing-off questionnare-9 (QUICK);^38^ and visual analog scales (VAS) for pain severity and frequency.^39^

### Ethical aspects

The study was approved by the respective hospital ethical committees/institutional review boards. In the United Kingdom, the study was adopted by the National Institute of Health Research Central Research Network (UKCRN No 13344).^10^ All participants provided informed consent before their entry to the study.

### Data analysis

Descriptive statistics (mean, standard deviation, median, range, percentages) were applied as needed. A single value for the applied VAS of pain (see Assessments) was obtained from (severity × frequency)/100. The frequency of each type of pain was determined considering each KPPS item score = 0 as ‘not present’ and ≥ 1 ‘present’.

Main variables in the study did not show a normal distribution (Shapiro-Francia test); therefore, non-parametric tests were used for comparison and correlation. The Mann–Whitney test was used to compare HRQoL indexes between patients with and without each type of pain assessed by the KPPS. The Benjamini–Hochberg correction for multiple comparisons was applied.^40^ Strength of the association was analyzed with the Spearman correlation coefficient and considered ‘moderate’ for coefficient values 0.35 to 0.50, and ‘strong” with values >0.50.

The influence of pain on patients’ HRQoLwas explored in two phases: (1) Influence of pain burden and types controlling for other relevant variables in the study, and (2) Influence of each specific syndromic pain contained in the KPPS. For phase 1, multiple linear regression models were constructed with the EQ-5D-3L and PDQ-8 indexes as dependent variables and age, sex, PD duration, SCOPA-Motor subscales (Activities of Daily Living, dyskinesias, and fluctuations), HADS-Depression, PDSS-2 total score, and KPPS total score or individual domains as explanatory variables. For phase 2, dependent variables were again EQ-5D-3L and PDQ-8 indexes, whereas the explanatory variables were age, sex, PD duration, and the KPPS domains. Normality of residuals, multicollinearity and homoscedasticity were checked and found acceptable in the four models.

## Electronic supplementary material

Supplementary material

Appendix
