# Supplementary material for: Distribution and impact on quality of life of the pain modalities assessed by the King’s Parkinson’s disease pain scale
Source: NPJ Parkinsons Dis. 2017 Mar 15;3:8. doi: 10.1038/s41531-017-0009-1 (PMC5459857; doi:10.1038/s41531-017-0009-1)
Supplement: Supplementary file 1 — Supplementary material [file 41531_2017_9_MOESM1_ESM.pdf]

Supplementary material

Table 1S – Demographic and historic data

	**Patients**
Gender –Male (%)	68.5
Age (years)*	64.38±11.38<br>(29 – 85)
Education (years)*	14.05±3.80<br>(9 – 24)
Duration of disease (years)*	5.40±4.93<br>(0 – 26)
Hoehn and Yahr stage (%)<br>HY stage 1<br>HY stage 2<br>HY stage 3<br>HY stage 4<br>HY stage 5	 21.4<br>47.75<br>23.60<br>6.74<br>0.56
Levodopa equivalent daily dose (mg.)*	539.49±435.77<br>(0 – 2065)
Patients with Motor dyskinesias (%)	32.6
Patients with Motor fluctuations (%)	45.5
Patients with Nocturnal akinesia (%)†	51.7

N= 178.

* Mean±SD (range).

† As per the item 9 of the Parkinson's Disease Sleep Scale-Version 2.

Table 2S - Correlation of the King's Parkinson´s Disease Pain Scale domains with the health-related quality of life measures

	**EQ-5D**	**PDQ-8**
1. Musculoskeletal pain	-0.36	0.20*
2. Chronic pain	-0.24*	0.38
3. Fluctuation-related pain	-0.38	0.37
4. Nocturnal pain	-0.30	0.42
5. Oro-facial pain	-0.26	0.32
6. Discolouration, oedema/swelling	-0.34	0.42
7. Radicular pain	-0.28	0.41
**KPPS Total score**	-0.56	0.58

All $p<0.001$, except (*) $p<0.01$
