# Supplementary material for: Distribution and impact on quality of life of the pain modalities assessed by the King’s Parkinson’s disease pain scale
Source: NPJ Parkinsons Dis. 2017 Mar 15;3:8. doi: 10.1038/s41531-017-0009-1 (PMC5459857; doi:10.1038/s41531-017-0009-1)
Supplement: Supplementary file 2 — Appendix [file 41531_2017_9_MOESM2_ESM.docx]

**Appendix**

Members of the KPPS, EUROPAR and the IPMDS Non Motor PD Study Group:

O. Rascol, University Hospital of Toulouse, and University of Toulouse 3, Toulouse, France; S. Pal, Forth Valley Royal Hospital, Scotland, UK.; D. Martino, Lewisham& Greenwich NHS Trust, London, UK; C. Carroll, Plymouth University and Plymouth Hospitals NHS Trust, Plymouth, UK; D. Paviour, St Georges Hospital, London, UK; C. Falup-Pecurariu, Transilvania University, Brasov, Romania; B. Kessel, Princess Royal University Hospital site, King's College Hospital, Orpington, UK; M. Silverdale, Greater Manchester Neuroscience Centre, Manchester, UK; A. Todorova, King's College Hospital, London, UK.
